# Effects of Botulinum Toxin Type A on Pain among Trigeminal Neuralgia, Myofascial Temporomandibular Disorders, and Oromandibular Dystonia

**DOI:** 10.3390/toxins13090605

**Published:** 2021-08-29

**Authors:** Kazuya Yoshida

**Affiliations:** Department of Oral and Maxillofacial Surgery, National Hospital Organization, Kyoto Medical Center, 1-1 Mukaihata-cho, Fukakusa, Fushimi-ku, Kyoto 612-8555, Japan; yoshida.kazuya.ut@mail.hosp.go.jp; Tel.: +81-75-641-9161; Fax: +81-75-643-4325

**Keywords:** botulinum toxin type A, trigeminal neuralgia, orofacial pain, temporomandibular disorders, myofascial pain, oromandibular dystonia, botulinum toxin therapy

## Abstract

The differences in analgesic effects of botulinum toxin type A were compared in 28 patients with trigeminal neuralgia, 53 patients with myofascial temporomandibular disorders, and 89 patients with the jaw closing oromandibular dystonia. The patients were treated by injection of botulinum toxin type A into the masseter, temporalis, medial pterygoid, and other muscles based on the symptoms of each patient. The pain severity was evaluated using the visual analog scale, pain frequency, and pain scale of the oromandibular dystonia rating scale. Botulinum toxin injection was performed 1068 times in all patients without significant adverse effects. The visual analog, pain frequency, and pain scales at baseline were reduced (*p* < 0.001) after two, four, eight, and 12 weeks after the first botulinum toxin therapy and at the endpoint. The effects differed significantly (*p* < 0.001) among the groups (repeated-measures analysis of variance). The mean improvement (0%, no effect; 100%, complete recovery) at the endpoint was 86.8% for trigeminal neuralgia, 80.8% for myofascial pain, and 75.4% for oromandibular dystonia. Injection of the botulinum toxin can be a highly effective and safe method to treat trigeminal neuralgia, myofascial pain, and oromandibular dystonia.

## 1. Introduction

The botulinum toxin, which can exert a paralytic effect by binding to presynaptic cholinergic nerve terminals at the neuromuscular junction, is produced by the Gram-positive, rod-shaped, spore-forming, anaerobic bacterium *Clostridium botulinum* [1]. It internalizes and inhibits the exocytosis of the neurotransmitter acetylcholine by decreasing the frequency of acetylcholine release. Consequently, it was originally applied for treating diseases associated with muscle hyperactivity, such as dystonia [2,3,4,5]. The toxin has also been used for diseases in the orofacial region, such as oromandibular dystonia [6,7,8,9,10,11,12], trigeminal neuralgia [13,14,15,16,17,18,19,20], temporomandibular disorders, and bruxism [21,22,23,24,25,26,27,28,29,30,31]. Furthermore, the botulinum neurotoxin has been proven to be effective for pain relief of different pain types, including chronic migraine, neuropathic pain, trigeminal neuralgia, and myofascial pain [32,33,34]. However, the mechanism underlying its analgesic effects remains uncertain. Randomized controlled studies have produced controversial results [22,23,24,25,26,29,31].

Trigeminal neuralgia (TN) is characterized by sudden, unilateral, electric shock-like pain that is limited to the distribution of one or more divisions of the trigeminal nerve [35]. Several authors have reported that subcutaneous or submucosal injection of botulinum toxin type A (BoNT/A) into the trigger zone may be an effective treatment option for patients with TN [13,14,15,16,17,18,19,20].

Temporomandibular disorders (TMDs) are a heterogeneous group of chronic pain conditions that affect the temporomandibular joint, masticatory muscles, and related structures. Despite the inconsistency of published data concerning the application of persistent myofascial pain with BoNT/A [25,26,27,28,29,30,31], shortcomings such as a small sample size and a lack of standardized management protocols have led to inconsistent results.

Dystonia is a movement disorder characterized by sustained, intermittent, or task-specific muscle contractions that result in abnormal repetitive movements and/or postures [36]. Oromandibular dystonia (OMD) is a focal type of dystonia involving the masticatory, lingual, and/or muscles of the stomatognathic system [8,10,11,12,37,38,39]. Symptoms related to OMD include masticatory disturbance, restricted mouth opening capacity, muscle pain, dysphagia, dysarthria, esthetic problems, life-threatening upper airway obstructions [8], and temporomandibular joint dislocations [8,40]. Most of these symptoms may cause impaired activities of daily living, cosmetic disfigurement, and social embarrassment and negatively impact the patient’s quality of life [38]. This study aimed to compare the analgesic effects, safety, and difference of BoNT/A on pain in TN, TMD with masticatory myofascial pain, and OMD.

## 2. Results

### 2.1. Patient Characteristics

The demographic characteristics of the patients with TN, myofascial TMD, and OMD are shown in Table 1. Mean age was significantly higher for TN (68.2 ± 13.6 years) than for myofascial TMD (46.1 ± 17.6 years, *p* < 0.001) and OMD (56.4 ± 16.2 years, *p* < 0.001). Patients with OMD were significantly (*p* < 0.003) older than those with myofascial TMD (Table 1). The three patient groups showed female preponderance, and the male/female ratio among the groups showed no statistically significant differences (Table 1). The number (percent) of patients with a history of psychiatric diseases is shown in Table 1. Psychiatric diseases included depression, schizophrenia, and mood disorders.

Only one of the 28 cases with TN had bilateral TN; the other 27 had unilateral TN. Sixteen patients had TN in the third division, 12 cases had TN in the second division, and one had TN in both the second and third divisions.

Forty-two patients with TMD (79.2%) showed bilateral symptoms, while 11 patients (20.8%) had unilateral symptoms. Symptomatic muscles included the masseter, temporalis, medial pterygoid, lateral pterygoid, posterior belly of the digastric, and sternocleidomastoid muscles.

Eighty-one patients with OMD (91.0%) showed bilateral symptoms, while eight patients (9.0%) had unilateral symptoms.

Symptomatic muscles included the masseter, temporalis, medial pterygoid, lateral pterygoid, orbicularis oris, posterior belly of digastric, sternocleidomastoid, risorius, mentalis, genioglossus, platysma, anterior belly of digastric, depressor anguli, trapezius, and zygomatic major muscles.

### 2.2. Treatment Outcomes

BoNT/A (Botox, Allergan, Irvine, CA, USA) injections were performed 1068 times for all patients. Adverse effects included tenderness and pain at the injection site and temporary muscle weakness after BoNT/A injection. These side effects disappeared spontaneously, and no major side effects were observed. The results of the visual analog scale, pain frequency, and pain scale of the oromandibular dystonia rating scale at baseline the after lidocaine and BoNT/A injections are summarized in Table 2, Table 3 and Table 4. The mean improvement of the visual analogue scale was significantly higher (*p* < 0.001) in patients without psychiatric treatment (*n* = 108, 81.0%) than with it (*n* = 61, 75.1%). A significant (*p* < 0.005) negative correlation (−0.238) was observed between the number of injected sites and improvement of the visual analog scale at the endpoint. The pain frequency (*p* = 0.155) and pain scales of the oromandibular dystonia rating scale (0.244) showed no significant correlations. The mean follow-up duration was 24.2 ± 9.1 months.

#### 2.2.1. Visual Analog Scale

**Figure 1:**
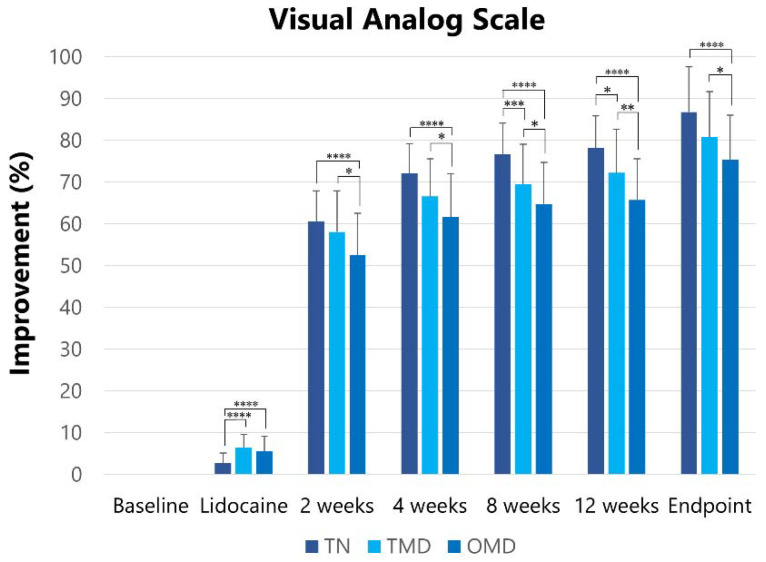
Changes in improvements in visual analog scale scores. Changes in the degree of improvement of visual analog scale scores were observed after lidocaine injection and at two, four, eight, and 12 weeks after BoNT/A injections. TN: trigeminal neuralgia, TMD: temporomandibular disorder, OMD: oromandibular dystonia. * *p* < 0.05, ** *p* < 0.01, *** p < 0.005, **** p < 0.001.

The visual analog scale at baseline was reduced (*p* < 0.001) after the lidocaine injection, at two, four, eight, and 12 weeks after the first botulinum toxin therapy, and at the endpoint (Table 2). The visual analog scale score at baseline was significantly (*p* < 0.001) higher in TN (89.3 ± 7.5) than in TMD (70.2 ± 19.3) and OMD (70.3 ± 19.1) patients (Table 2). Therefore, changes (%) in the visual analog scale after treatment were compared among the groups as a primary outcome measure. A significant difference was found in Mauchly’s sphericity test. Therefore, the Greenhouse–Geisser correction was performed to adjust for the lack of sphericity in a repeated measures ANOVA. The effects were significantly different among the groups (*F* = 13.568, *p* < 0.001). A post hoc test was performed at each follow-up. Significant differences are shown in Figure 1.

#### 2.2.2. Pain Frequency

**Figure 2:**
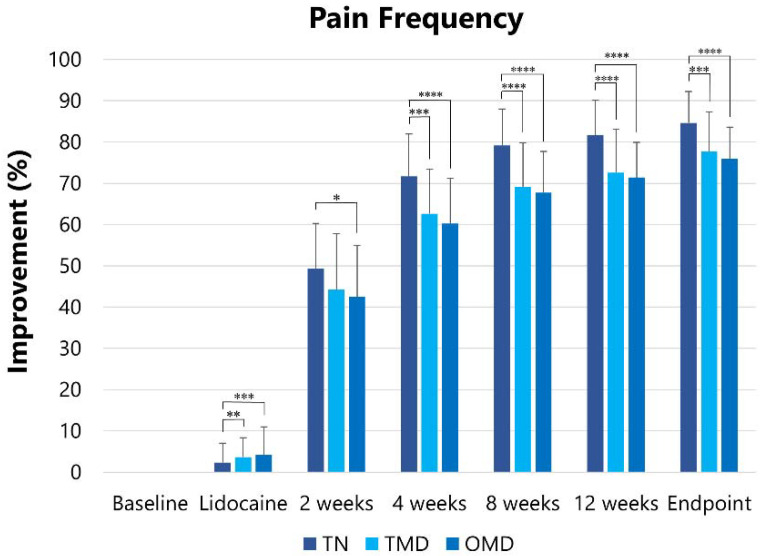
Changes in improvement in pain frequency. Changes in the degree of improvement in pain frequency were observed after the lidocaine injection and at two, four, eight, and 12 weeks after BoNT/A injections. TN: trigeminal neuralgia, TMD: temporomandibular disorders, OMD: oromandibular dystonia. * *p* < 0.05, ** *p* < 0.01, *** p < 0.005, **** p < 0.001.

The pain frequency at baseline was reduced (*p* < 0.001) after lidocaine injection, two, four, eight, and 12 weeks after the first botulinum toxin therapy, and at the endpoint (Table 3). The pain frequency at baseline was significantly higher in TN (19.1 ± 7.7) than in TMD (13.8 ± 6.8, *p* < 0.005) and OMD (12.5 ± 5.7, *p* < 0.001). The Greenhouse–Geisser correction was performed to adjust for the lack of sphericity. The effects were significantly different among the groups (*F* = 4.036, *p* < 0.005). The significant differences analyzed by the post hoc test are shown in Figure 2.

#### 2.2.3. Pain Scale of the Oromandibular Dystonia Rating Scale (OMDRS)

**Figure 3:**
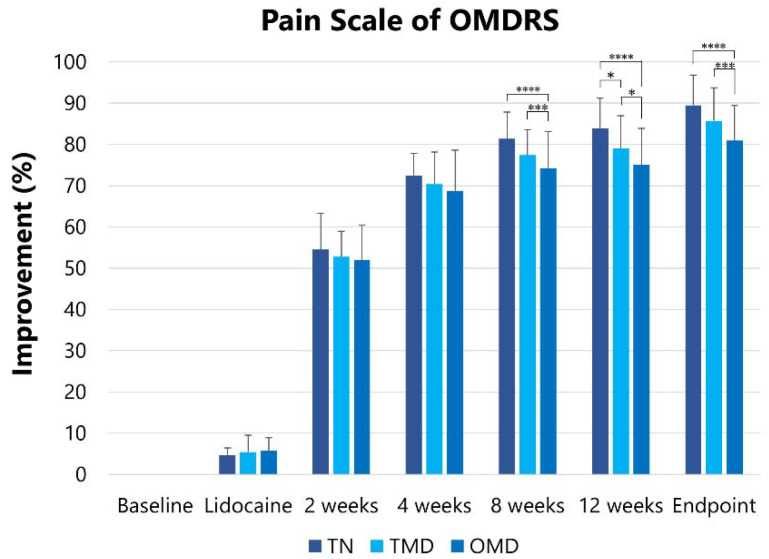
Changes in improvement in the pain scale score of the OMDRS. Changes in the degree of improvement in the pain scale score of the OMDRS are shown after injection and at two, four, eight, and 12 weeks after BoNT/A injections. OMDRS: oromandibular dystonia rating scale, TN: trigeminal neuralgia, TMD: temporomandibular disorders, OMD: oromandibular dystonia. * *p* < 0.05, *** p < 0.005, **** p < 0.001.

The pain scale at baseline was reduced (*p* < 0.001) after the lidocaine injection, two, four, eight, and 12 weeks after the first botulinum toxin therapy, and at the endpoint (Table 4). The pain scale of the OMDRS [38] at baseline was significantly (*p* < 0.001) higher in TN (28.6 ± 3.6) than in TMD (21.6 ± 7.5) and OMD (20.3 ± 7.4). After the Greenhouse–Geisser correction, the effects were significantly different among the groups (*F* = 18.777, *p* < 0.001). Significant differences are shown in Figure 3.

### 2.3. Trigeminal Neuralgia (TN)

In 16 patients with third-division TN, BoNT/A was injected submucosally or subcutaneously into the trigger zone. Twelve patients with second-division TN required sphenopalatine block using a customized computer-aided design/computer-aided manufacturing (CAD/CAM)-derived needle guide [41]. BoNT/A was injected a total of 136 times (mean, 4.9 ± 2.5 times; range, 1–8 times). The average dose per injection was 43.1 ± 5.3 units. The mean improvement in the visual analog scale score was significantly (*p* < 0.05) higher in patients injected using the CAD/CAM needle guide (*n* = 12, 91.2%) than in those that were not (*n* = 16, 82.9%).

### 2.4. Myofascial Temporomandibular Disorders (TMD)

BoNT/A was injected a total of 195 times (mean: 3.7 ± 4.1 times, range: 1–18 times) without any adverse effects. The average dose per injection into the muscles was 56.3 ± 7.2 units. The injected jaw-closing muscles were the masseter (92.5%), temporalis (58.5%), and medial pterygoid (32%) muscles. Other injected muscles included the lateral pterygoid (30.2%), posterior belly of the digastric (24.5%), and sternocleidomastoid muscles (18.9%).

### 2.5. Oromandibular Dystonia (OMD)

BoNT/A was injected a total of 737 times (mean: 4.4 ± 4.0 times, range: 1–20 times) without any significant complications. The average dose per injection into the muscles was 58.2 ± 6.7 units. Injected muscles were the masseter, temporalis, and medial pterygoid, each at 85.4%, 52.8%, and 10.1%, respectively. Other injected muscles were the lateral pterygoid (18%), orbicularis oris (6.7%), posterior belly of digastric (6.7%), sternocleidomastoid (5.6%), risorius (4.5%), mentalis (4.5%), genioglossus (4.5%), platysma (3.4%), anterior belly of digastric (2.2%), depressor anguli (1.1%), trapezius (1.1%), and zygomatic major (1.1%) muscles.

## 3. Discussion

The present study is the first to compare the differences in analgesic effects of BoNT/A on pain among TN, myofascial TMD, and OMD subjects.

### 3.1. Mechanism of BoNT/A on Muscle Relaxation and Pain Relief

The botulinum toxin has been studied as a promising option for the treatment of various types of pain [32,33,34]. The mechanism of its pain-relieving action is not entirely clear; however, it is believed to inhibit neurotransmitter release from primary sensory neurons, thus inhibiting peripheral and central sensitization [42,43].

Synaptic vesicles, which contain neurotransmitters, dock and fuse with the plasma membrane through interactions with soluble *N*-ethylmaleimide-sensitive factor attachment protein receptor proteins (synaptobrevin, synaptosomal-associated protein-25, and syntaxin). BoNT/A binds to the membrane and a receptor at the neuromuscular junction, followed by internalization into endosomes. Subsequently, the light chain translocates across the membrane into the cytosol. The light chain cleaves synaptosomal-associated protein-25, which inhibits acetylcholine release at the motor endplate.

Animal studies have proven that subcutaneous BoNT/A inhibits formalin-induced pain by preventing the release of inflammatory mediators such as glutamate, substance P, and calcitonin gene-related peptides from nociceptive nerve endings [44,45]. BoNT/A had not only peripheral effects, such as decreasing levels of substance P, calcitonin gene-related peptide, and glutamate [44], but also central effects, such as axonal transport to the sensory regions of the trigeminal ganglia on sensitive neurotransmitters or sensory nerves [42,43].

### 3.2. Limitations of This Study

This study had several limitations. In it the retrospective results of botulinum toxin therapy for pain were compared among the three groups. No control group was set up for ethical reasons. The author has launched a website on involuntary movements of the stomatognathic system [46]. Many patients, including those with TN, were referred to the author over long distances [47]. In Japan, botulinum toxin therapy for TN, masticatory myofascial pain, and OMD can only be clinically applied in our clinic, therefore, patients with high expectations often must travel very long distances to visit our department. For this reason, a control group could not be set from an ethical point of view.

The difficulty in objectively assessing pain has already been discussed [48,49]. Many authors have evaluated pain using a visual analogue scale or pain frequency. However, it is very difficult to comprehensively evaluate the severity and changes in pain. Pain can be influenced by many factors, including psychiatric factors. Since the pain parameters used in this study (visual analog scale, pain frequency, and OMDRS) can be influenced considerably by a placebo effect, the results should be interpreted carefully. Furthermore, the placebo effect associated with the new method cannot be excluded from this study.

In this study, the results were compared at baseline and after lidocaine and BoNT/A administration. Before botulinum toxin therapy, local anesthetic was injected into the muscles, which are postulated to be the cause of pain or involuntary movements. The procedure can help to predict the effect of the botulinum toxin therapy and whether the muscles are the cause of patients’ symptoms. If the symptoms disappear, even if only during the anesthetic effect, the injected muscles must be the cause of the symptoms, and botulinum toxin will help [39]. On the contrary, if the procedure shows no effect; there must be other causes for the patients’ symptoms, such as psychogenic factors. For such cases, BoNT/A injections were not administered. Non-responders to BoNT/A must be excluded from this step. This procedure is an improvement in this study compared to previous studies. Although the change after the lidocaine injection was slight, significant differences between baseline and after the injections were observed (Table 2).

Women were more prevalent than men in all three types of diseases analyzed in this study. However, the age of onset was the youngest in the TMD group and the highest in the TN group. In other words, TMD is more common in individuals in their 20s and 30s, OMD is normally observed in those in their 50s and older, and TN is seen in those in their 60s and older. In this study, patients with TMD were older than the average age of patients with general TMD because other conservative therapies have been ineffective for many years. However, there was a significant age difference among the three groups (Table 1).

Local injections of lidocaine and ethanol can block afferents in the muscle spindle. Muscle afferent block therapy was used effectively in patients with OMD and masticatory muscle spasms [50,51,52]. Currently, muscle afferent block therapy is widely used to predict the effectiveness of botulinum toxin therapy or to extend its effect between the intervals of the therapy. Dry needling and wet needling have been commonly applied for myofascial pain and dysfunction [53]. A recent meta-analysis concluded that the efficacy of needling therapy did not depend on the needling type or substance [54,55]. Further studies are necessary to clarify the mechanism underlying the effects of muscle afferent block therapy.

### 3.3. Effects of BoNT/A on TN

TN pain can occur after stimulation of the trigger zone. Although TN is most commonly related to microvascular compression, the pathophysiological mechanism underlying its development is not fully understood [35]. Pharmacological therapy remains the first-line treatment; however, some cases may necessitate surgical procedures such as microvascular decompression, and gamma knife stereotactic radiosurgery [35]. However, surgical options are not always efficacious, and occasionally result in severe complications or symptom recurrence. Four double-blind randomized controlled trials reported that submucosal or subcutaneous injection of botulinum toxin was effective for patients with TN, and the results exhibited a significant benefit [17,18,19,20]. In most studies, a response was achieved in approximately 70–90% of patients, and the mean pain intensity and frequency were reduced by approximately 50–90% by four weeks after the injection. In the four studies, the botulinum toxin was injected subcutaneously, intradermally, or submucosally. Adverse effects include edema, hematoma, pain, facial asymmetry, and masticatory disturbances [13,14,15,16,17,18,19,20]. The last two complications can be related to their influences on the masseter muscle. Conversely, about 90% of patients responded by the sixth month in two open-labeled studies on botulinum neurotoxin therapy administered via sphenopalatine ganglion injection [56,57]. Sphenopalatine ganglion blocks have been used by clinicians in the treatment of various pain syndromes, such as cluster headaches, migraines, and TN. However, the mechanism of action remains uncertain. BoNT/A can limit the release of inflammatory mediators, thus counteracting central sensitization [45]. Further animal studies must be performed to clarify the mechanism. In the present report, BoNT/A injections were administered to 12 patients using CAD/CAM guides [41]. All patients responded without complications. It has been suggested that because of the low diffusion gradient of the neurotoxin, it may be required to inject sufficient doses closer to the sphenopalatine ganglion. Some authors have reported freehand insertion of the needle into the sphenopalatine ganglion region [56,57]. However, administering a sphenopalatine ganglion block requires adequate experience. Some researchers have also reported adverse effects such as bleeding, swelling, and epistaxis accompanying the injection [56,57,58]. The more times the needle is inserted into the region, the greater the risk of complications. Using the CAD/CAM-derived guide, the needle was inserted without any complications. This result may be related to the single insertion using the method. Even without prior experience with sphenopalatine ganglion blocks, the guide enables the accurate and safe administration of the botulinum toxin to the sphenopalatine ganglion [41].

### 3.4. Effects of BoNT/A on Myofascial TMD

The most common symptom in TMD is masticatory myofascial pain, which often refers to pain in the neck, face, or preauricular regions. It is often accompanied by headaches and limited mouth opening. This disorder has a complex pathogenesis and is expressed as a multifactorial etiology, as well as with various systemic and local risk factors [59]. A multimodal approach with conservative treatment is recommended such as counseling, pharmacotherapy, physiotherapy, and occlusal splints. This treatment regimen is often efficacious, but the pain does not resolve in some patients [27]. A randomized, placebo-controlled, crossover multicenter study showed no significant difference in pain relief between BoNT/A and saline injection into the masseter muscle of patients with persistent myofascial TMD [26]. Their results support previous findings from systematic reviews on myofascial pain conditions in other body parts [60,61]. Their results are also consistent with those of two randomized controlled studies on myofascial TMD [23,25]. Recent randomized controlled studies reported clear treatment effects of BoNT/A [29,31].

BoNT/A may have an analgesic effect which is independent of its muscle-relaxing effects, because pain reduction can precede muscle relaxation and last for a longer period, and it is also present outside injected sites. In a recent randomized clinical trial, BoNT/A revealed almost the same effect on myofascial pain as an occlusal splint [29]. Taking into consideration the higher costs compared to other conventional options, the contraindications for women who are pregnant or breastfeeding, and the side effects, BoNT/A will have very little benefit for patients. However, although the patients were older and had a longer disease duration than those in previous studies [25,26,29], the patients showed a much higher improvement in this study. Most studies have evaluated the single use of BoNT/A. In some patients with high expectations, the first injection of BoNT/A had a dramatic effect. However, in most patients, the effect is gradually improved by repeating the onset of the effect after injection and the diminishing effect over time. Therefore, it is clinically more meaningful to judge the effect when the symptoms are subsided, the patient is satisfied, and the botulinum toxin therapy is completed, rather than evaluating the effect of only a single treatment. Botulinum toxin therapy should only be applied in TMD cases where conventional methods are ineffective and should not be attempted without attempting general, reversible methods for myofascial TMD.

### 3.5. Effects of BoNT/A on OMD

Dystonia is manifested by sustained, intermittent muscle, or task-specific contractions that cause abnormal movements or postures [36]. OMD is a focal type of dystonia involving the masticatory and/or lingual muscles. OMD includes jaw closing dystonia, jaw opening dystonia, lingual dystonia, jaw deviation dystonia, jaw protrusion dystonia, and lip dystonia [37,38]. A recent study postulated that OMD has an equal or higher prevalence of cervical dystonia or blepharospasm [62]. The approaches to OMD treatment should be taken seriously not only by neurologists or neurosurgeons, but also by oral and maxillofacial surgeons or dentists [37,38,39,62]. Patients with OMD were successfully treated with BoNT/A. However, patients with severe trismus related to this disease, in whom treatment with botulinum neurotoxin injections, muscle afferent block therapy or sensory trick splint [63] had been insufficient, successfully underwent coronoidotomy [64,65,66]. This study focused on jaw closing dystonia associated with pain. Therefore, other subtypes of OMD were excluded from the analysis in this study.

Patients with OMD have very variable symptoms from patient to patient, such as masticatory disturbance, dysarthria, dysphasia, pain, and esthetic problems. In this study, task-specificity was observed in 49.4% of the patients, and muscle hyperactivity was observed only during specific tasks. Pain is not always the chief complaint in patients with OMD. Patients without complaints of pain were excluded from the study. Recently, the OMDRS has been developed and validated [38]. It can be useful for the comprehensive evaluation including disease severity, disability, psychosocial functioning, and impact on quality of life. This study focused on pain and posttreatment changes. In this study, only the subscale of pain on the examiner-rated scale (0–40 points) was evaluated.

### 3.6. Difference of Effects of BoNT/A among the Three Groups

BoNT/A has two main effects. The first is muscle relaxation, and the other is pain relief. In TMD, hyperactive masticatory muscles often result in pain in the muscles or temporomandibular joints. Patients with arthrogenous pain were excluded from the study. In OMD, dystonic contraction in the muscles of the stomatognathic system can result in pain. In the present study, only the jaw closing type of patients was analyzed to compare the results with those of patients with TMD. Based on the two effects of BoNT/A, it may be reasonable to consider that BoNT/A is more effective in patients with TMD or OMD than in those with TN. However, the results were contrary. Although the reason for the results is uncertain, a possible explanation might be more limited injection sites in TN than in TMD or OMD. The patients with TN in this study were unilateral, except for one patient. Only one case had symptoms in the second and third divisions of the trigeminal nerve. One 50-unit vial of BoNT/A was sufficient in all cases. Compared to the myofascial TMD and OMD groups, a sufficient amount of BoNT/A can be injected into the localized area. Indeed, a significant (*p* < 0.005) negative correlation (−0.238) was observed between the number of injected sites and improvement of the visual analog scale at the endpoint. Furthermore, in 12 patients, the author used a customized CAD/CAM-derived needle guide during the injection of BoNT/A to the sphenopalatine ganglion for the treatment of second-division TN [41]. Therefore, the improvement rate may have been higher than in the other two groups. Pain is the main symptom in patients with TN. On the other hand, patients with OMD and TMD showed highly variable symptoms. As a result, it cannot easily lead to pain improvement.

### 3.7. Adverse Effects of BoNT/A

Previously reported adverse effects of BoNT/A include temporary regional weakness, tenderness over the injection area, discomfort during chewing, asymmetric smile, reduction in the size of the muscle, paresthesia, difficulty swallowing, speech changes, swelling, bruising, facial asymmetry, transient edema, itching, and pain at the injection site. Most of the effects were transient and spontaneously disappeared. Many of these adverse effects cannot occur with accurate knowledge of the local anatomy of muscles, nerves, and other tissues and accurate injection procedures. Most of these side effects are considered to be related to the injection technique. The more accurately the botulinum neurotoxin is injected into the target muscles, the more likely the improvement in the patient’s symptoms, and the lower the risk of adverse effects. BoNT/A injections into the masseter and temporal muscles are the simplest of the orofacial muscle locations compared to the tongue [12], lateral pterygoid [11,40], and medial pterygoid muscles [11]. Injection techniques as inconsistencies and limitations in previous reports have not been discussed so far, but it is quite possible that injection techniques due to empirical differences are associated with side effects. Previous studies did not describe how much experience the doctors performing the injections had. In a study [26], the authors learned the injection technique from a neurologist with expertise in BoNT/A injections before the study started. They practiced the injection technique using only two patients. The author of the present study has over 30 years of experience in treating patients with the botulinum toxin and has experienced very few side effects in the masseter and temporal muscles. It may be reasonable that there are considerable differences in therapeutic improvements and adverse effects between inexperienced and experienced clinicians.

It may be a shortcoming to consider the effectiveness or adverse effects of a single treatment by an inexperienced physician. However, tenderness and pain at the injection site associated with the injection itself and temporary muscle weakness after the response to BoNT/A are inevitable. These side effects should be explained to the patients in advance.

Several researchers have reported changes in the mandibular bone after BoNT/A injections in animals [67,68,69] and humans [70,71]. They claimed that the changes were an adverse effect. In contrast, a retrospective study in women with squared faces found no significant difference in the whole mandible volume and the cortical thickness of the mandibular ramus [72]. The changes in bone after BoNT/A cannot be an adverse effect, but a normal physiological response related to a decrease in masticatory force. Long-lasting forceful bruxism can result in a “square mandible” configuration with a coronoid process and angle hyperplasia [73]. The author believes that the injection of BoNT/A improves the excessive tension of the jaw closing muscles and allows the hypertrophied muscles and bones to return to their original shape. Recently, in esthetic dentistry, the botulinum toxin has been applied for masseter muscle hypertrophy to improve esthetics. The occlusal force is measured before and after each injection of BoNT/A and the treatment course is carefully observed so that there is no excessive decrease in occlusal force. In the author’s 30 years of clinical experience, none of the cases had any adverse events associated with bone resorption, such as bone fracture or osteoporosis.

### 3.8. Future Study on the Clinical Use of BoNT/A

In the future, randomized controlled trials with larger sample sizes and longer follow-up periods are necessary to determine the therapeutic effectiveness, optimal dose, duration of effect, adverse effects, and indications for botulinum toxin therapy. It is crucial to differentially diagnose patients for indications for BoNT/A injection. BoNT/A is expensive. It is important to predict and exclude non-responders for economic reasons. The discrepant results and adverse effects of previous studies may be related to injection techniques. Experienced physicians should inject BoNT/A at an adequate dose, possibly individually for each patient. Furthermore, comprehensive evaluation of not only pain but also associated symptoms and quality of life is required.

## 4. Conclusions

Injection of botulinum toxin type A can be a highly effective and safe way to treat TN, myofascial TMD, and OMD.

## 5. Materials and Methods

### 5.1. Patients

The demographic characteristics of the patients (TN, myofascial TMD, and OMD) are summarized in Table 1. Women who were pregnant, breastfeeding, or not on birth control were excluded. All patients were naïve to BoNT/A.

#### 5.1.1. TN

Twenty-eight patients (23 women and 5 men; mean age, 68.2 ± 13.6 years) with classical TN based on the beta version of the third edition of the International Classification of Headache Disorders [35] were enrolled in this study. The patients had a consultation in the Department of Neurosurgery and underwent magnetic resonance imaging, which did not detect any structural pathology or suggest the need for surgical procedures.

#### 5.1.2. Myofascial TMD

TMD involves a number of clinical conditions of the temporomandibular joint, masticatory muscles, and related structures. TMD was diagnosed based on the diagnostic criteria for TMD (DC/TMD) [74]. Fifty-three patients (39 women and 14 men; mean age, 46.1 ± 17.6 years) with persistent myofascial TMD without arthrogenous pathology, who were treated by conventional treatments (medications, oral appliances, or physical therapy) without sufficient improvement. Patients with temporomandibular joint pain (with or without reduction) were excluded.

#### 5.1.3. OMD

OMD was diagnosed according to the characteristic clinical features of focal dystonia and electromyographic findings [37,38,75]. The patients’ clinical features were stereotypy, task specificity, co-contraction, and morning benefit. Eighty-nine patients (60 women and 29 men; mean age, 56.4 ± 16.2 years) with jaw closing type OMD were enrolled in this study. Forty-four (49.4%) patients showed task specificity in which symptoms occurred only during specific tasks. These tasks included speaking (37.3%), chewing (21.3%), swallowing (2.2%), and mouth opening (2.2%). Other subtypes (jaw opening dystonia, jaw deviation dystonia, jaw protrusion dystonia, lingual dystonia, and lip dystonia) were excluded from the analysis in this study. Furthermore, patients with jaw closing dystonia without pain symptoms were excluded from this study.

The patients received an explanation of the treatment plan and provided written informed consent. This study was performed in accordance with the Declaration of Helsinki after obtaining approval from the institutional review board and ethics committee of the Kyoto Medical Center (15-031).

### 5.2. Botulinum Toxin Therapy

Before the BoNT/A was injected, 3–5 mL of 0.5% lidocaine (Xylocaine, Aspen Japan, Tokyo, Japan) was injected into the hyperactive or painful muscles in patients with TMD and OMD and into trigger points in patients with TN. Changes in pain and involuntary movements were carefully observed after the lidocaine injection. If the patients showed changes at all, no BoNT/A injections were administered. If the patients showed improvement of symptoms under the effects of the local anesthetic, we made their next appointments for the BoNT/A injections. Some patients claimed exacerbation of pain at the injection site after the lidocaine injection, which may be related to the injection itself. Pain was evaluated during the next visit.

BoNT/A was reconstituted with isotonic sodium chloride solution to reach a concentration of 2.5 to 5 units/0.1 mL. A disposable hypodermic needle electrode (TECA MyoJect Luer Lock, 37 mm × 25 G, 50 mm × 25 G, Natus Neurology, Middleton, WI, USA) was inserted into the target muscles [10,11,12]. The correct placement of the tip of the needle electrode was confirmed under EMG guidance using an EMG instrument (Neuropack n1, MEM-8301, Nihon Kohden, Tokyo, Japan) [10,11,12]. Subsequently, after aspiration, 10 to 25 units of BoNT/A were injected into the target muscles.

#### 5.2.1. TN

For patients with third-division TN, BoNT/A was injected submucosally or subcutaneously into the trigger zone. A CAD/CAM-derived injection guide was used for patients with second-division TN [41]. The guide was fabricated as follows. CT scans were performed on the patients while occluding their incisors on an elastic bite block; this was to avoid overlapping with the dental images of the opposing arches. The CT data (slice width, 0.5 mm) were stored in the ‘digital imaging and communications in medicine’ format [41]. The target point around the pterygopalatine fossa was determined after combining the CT data with a scan of a maxillary model using a soft program for dental implant surgery [41]. Fifty units of BoNT/A were reconstituted with 1 mL of isotonic sodium chloride solution. After gargling with a solution of Neostelin Green 0.2% mouthwash solution (Nippon Shika Yakuhin, Yamaguchi, Japan), the guide was affixed to the patient’s maxilla, and a disposable needle (60 mm × 23 G, Terumo, Tokyo, Japan) was inserted through a metal sleeve to the analyzed depth [41]. The guides were cleaned with an ultrasonic cleaner before being stored to allow for re-sterilization and to be used for subsequent injections.

#### 5.2.2. Myofascial TMD and OMD

For patients with TMD and OMD, the author administered 50 units of BoNT/A that were reconstituted with 1–2 mL of isotonic sodium chloride solution into the masseter, temporal, and medial pterygoid muscles [11,12] based on the patient’s symptoms. The maximum occlusal force was measured on the bilateral molars using an occlusal force meter (GM10, Nagano Keiki Co.; Tokyo, Japan). Muscles and doses of BoNT/A were individually determined for each patient based on their symptoms and occlusal force. If the author decided that 50 units were not enough, a 100-unit vial was used. The injection was continued until the patient was satisfied with the therapeutic effect, and then the treatment was completed. The injection interval was 3–6 months, depending on the patient’s symptoms and requests.

### 5.3. Analysis

Pain severity was evaluated using the visual analog scale, pain frequency, and OMDRS [38]. The visual analog scale was evaluated on a 100-mm line with the endpoints “no pain” and “worst imaginable pain.” The patients were requested to maintain a pain diary during treatment. The patients were also evaluated using the examiner-rated subscale of pain (Table 5) on the OMDRS [38]. Visual analog scale, pain frequency, and pain scale were evaluated at baseline and at two weeks, four weeks, eight weeks, and 12 weeks after the botulinum neurotoxin injection. The results obtained before and after BoNT/A therapy were compared. The degree of improvement (%) was calculated according to the following formula: (pre-treatment score − post-treatment score)/pre-treatment score (%), where 0% represents no improvement and 100% indicates complete recovery. The results were obtained at baseline, after local anesthetic injection, at two, four, eight, and 12 weeks after the BoNT/A injection, and at the endpoint. The endpoint was the time when the patient was satisfied with the therapeutic effect and the botulinum toxin therapy was completed. If pain reappeared, the same injection was repeated within at least 12 weeks intervals, and then the scores were recorded. Adverse events were recorded during each visit.

A two-tailed paired *t*-test was used. Differences among the three groups were statistically compared using one-way analysis of variance. Fisher’s exact test and unpaired *t*-tests were applied to assess the statistical significance of the differences in the distributions. All analyses were conducted using the statistical software package SPSS for Windows (version 14.0; SPSS Japan Inc. Tokyo, Japan). The null hypothesis was rejected at the 5% level (*p* < 0.05). Improvements and changes in the visual analog scale, pain frequency, and pain scales of the OMDRS were statistically analyzed with 2-way repeated measures analysis of variance (ANOVA) with the groups (TN, TMD, and OMD) and times (at baseline, after lidocaine injection, at two weeks, four weeks, eight weeks, and 12 weeks, and at the endpoint) as factors. When the sphericity was significant, the Greenhouse–Geisser correction was used to adjust for the lack of sphericity in the repeated measure ANOVA. The Bonferroni method was used as a post-hoc test when the ANOVA revealed significant differences.

## Figures and Tables

**Table 1:** Demographic characteristics of patients with each disease and the entire patient cohort.

Disease Groups	N	Age (Years) [Mean (SD)]	Sex (Female, Male) [N (%)]	Duration (Years) [Mean (SD)]	Visual Analog Scale at Baseline [Mean (SD)]	Pain Frequency at Baseline (Times/Day) [Mean (SD)]	Pain Scale of Oromandibular Dystonia Rating Scale (OMDRS) (Points) [Mean (SD)]				Psychiatric Disease [N (%)]
							Severity	Duration	Degree	Total	
TN	28	68.2 (13.6)	23 (82.1), 5 (17.9)	6.3 (5.6)	89.3 7.5)	19.1 (7.7)	18.9 (5.1)	4.8 (1.5)	4.9 (1.4)	28.6 (3.6)	4 (14.3)
TMD	53	46.1 (17.6)	39 (73.6), 14 (26.4)	10.1 (6.5)	71.3 (19.3)	13.8 (6.8)	10.9 (5.9)	3.6 (1.2)	3.5 (1.1)	21.6 (7.5)	17 (32.1)
OMD	89	56.4 (16.2)	60 (67.4), 29 (32.6)	3.7 (4.5)	70.3 (19.1)	12.5 (5.7)	10.4 (5.2)	3.5 (1.3)	3.1 (1.0)	20.3 (7.5)	40 (44.9)
Total	170	55.2 (17.9)	122 (71.8), 48 (28.2)	4.9 (5.5)	73.4 (19.1)	14.0 (6.8)	11.6 (5.6)	3.7 (1.2)	3.4 (1.1)	22.1 (7.6)	61 (35.9)

TN, trigeminal neuralgia; TMD, temporomandibular disorder; OMD, oromandibular dystonia; SD, standard deviation.

**Table 2:** Results of the botulinum toxin therapy on the visual analog scale.

Groups	Visual Analog Scale [Mean (SD)]						
	Baseline	After Lidocaine Injection	After BoNT/A Injection				
			2 Weeks	4 Weeks	8 Weeks	12 Weeks	Endpoint
TN	89.3 (7.5)	86.8 (7.3)	35.1 (6.6)	25.0 (6.8)	20.8 (7.0)	19.5 (7.3)	11.9 (9.8)
TMD	71.2 (19.3)	65.6 (18.1)	28.8 (9.4)	23.2 (8.9)	21.2 (9.2)	19.8 (9.8)	14.2 (9.2)
OMD	70.0 (19.1)	64.5 (17.7)	32.4 (8.2)	26.0 (7.5)	24.0 (7.8)	23.5 (8.0)	17.3 (8.5)
Total	73.4 (19.1)	66.3 (17.6)	31.7 (8.6)	25.0 (7.9)	22.6 (8.2)	21.7 (8.6)	15.4 (9.1)

After BoNT/A injection, visual analog scale scores had reduced significantly (*p* < 0.001) at two, four, eight, and 12 weeks from baseline. TN, trigeminal neuralgia; TMD, temporomandibular disorder; OMD, oromandibular dystonia; SD, standard deviation.

**Table 3:** Results of the botulinum toxin therapy by pain frequency.

Groups	Pain Frequency (Times/Day) [Mean (SD)]						
	Baseline	After Lidocaine Injection	After BoNT/A Injection				
			2 Weeks	4 Weeks	8 Weeks	12 Weeks	Endpoint
TN	19.1 (7.7)	18.6 (7.5)	9.8 (4.9)	5.6 (3.5)	4.2 (2.9)	3.7 (2.6)	3.1 (2.3)
TMD	13.8 (6.8)	13.2 (6.5)	8.0 (4.7)	5.3 (3.2)	4.3 (2.6)	4.0 (2.6)	3.3 (2.3)
OMD	12.5 (5.7)	12.0 (5.7)	7.4 (4.0)	5.1 (2.9)	4.0 (2.3)	3.6 (2.0)	3.0 (1.7)
Total	14.0 (6.8)	13.5 (6.7)	8.0 (4.5)	5.3 (3.1)	4.2 (2.5)	3.7 (2.3)	3.1 (2.0)

After BoNT/A injection, pain frequency had reduced significantly (*p* < 0.001) at two, four, eight, and 12 weeks from baseline. TN, trigeminal neuralgia; TMD, temporomandibular disorder; OMD, oromandibular dystonia; SD, standard deviation.

**Table 4:** Results the botulinum toxin therapy on the pain scale of the oromandibular rating scale.

Groups	Pain Scale of Oromandibular Dystonia Rating Scale (OMDRS) (Point) [Mean (SD)]						
	Baseline	After Lidocaine Injection	After BoNT/A Injection				
			2 Weeks	4 Weeks	8 Weeks	12 Weeks	Endpoint
TN	28.6 (3.6)	27.3 (3.5)	13.1 (3.3)	7.8 (1.5)	5.3 (1.9)	4.6 (2.3)	3.1 (2.3)
TMD	21.6 (7.5)	20.4 (7.0)	10.3 (4.1)	6.5 (3.0)	4.9 (2.2)	4.6 (2.4)	3.3 (2.2)
OMD	20.3 (7.5)	19.1 (7.1)	9.8 (4.2)	6.4 (3.2)	5.3 (2.8)	5.1 (2.8)	3.9 (2.3)
Total	22.1 (7.6)	20.4 (7.3)	10.5 (4.2)	6.7 (2.9)	5.2 (2.5)	4.9 (2.6)	3.6 (2.3)

After BoNT/A injection, the pain scale score of the OMDRS had reduced significantly (*p* < 0.001) at two, four, eight, and 12 weeks from baseline. TN, trigeminal neuralgia; TMD, temporomandibular disorder; OMD, oromandibular dystonia; SD, standard deviation.

**Table 5:** Examiner-rated pain scale of the OMDRS [38].

1. Rate the severity of pain during the last week on a scale of 0 to 10, where a score of 1 represents a minimal ache and 10 represents the most excruciating pain imaginable.	
Best	0 to 10
Worst	0 to 10
Usual	0 to 10
2. Rate the duration of pain	
None	0
Present < 10% of the time	1
Present 10% to <25% of the time	2
Present 25% to <50% of the time	3
Present 50% to <75% of the time	4
Present ≥ 75% of the time	5
3. Rate the degree to which pain contributes to disability	
No limitation or interference from pain	0
Pain is quite bothersome but not a source of disability	1
Pain definitely interferes with some tasks a major contributor to disability	2
Pain accounts for some (less than half) disability	3
Pain is a major source of difficulty with activities; separate from this, muscle contraction is also a source of some (less than half) disability	4
Pain is the major source of disability; without it, most impaired activities could be performed quite satisfactorily	5
4. Total Pain Score	

Total score is the sum of 1–3 (maximum score, 40).

## Data Availability

The raw data supporting the conclusions of this article will be made available by the author, without undue reservation, to any qualified researcher.
